# Study on Delamination Damage of CFRP Laminates Based on Acoustic Emission and Micro Visualization

**DOI:** 10.3390/ma15041483

**Published:** 2022-02-16

**Authors:** Wei Li, Yinghonglin Liu, Peng Jiang, Fuping Guo, Jiahao Cheng

**Affiliations:** 1 College of Mechanical Science and Engineering, Northeast Petroleum University, Daqing 163318, China; liweinepu@163.com (W.L.); jpnepu@163.com (P.J.); gfpmmc@163.com (F.G.); wojiaochengjiah@163.com (J.C.); 2 College of Mechanical and Electrical Engineering, Guangdong University of Petrochemical Technology, Maoming 525000, China

**Keywords:** acoustic emission, multidirectional laminate, delamination damage, strain energy release rate, DCB

## Abstract

This study investigated the mechanism of delamination damage in the double cantilever beam (DCB) standard test by the use of the strain energy release rate. The curve of the strain energy release rate was verified by the Rise Angle (*RA*) method. For this purpose, 24-layer carbon fiber/epoxy multidirectional laminates with interface orientations of 0°, 30°, 45°, and 60° were fabricated according to the standard ASTM D5528**(13)**. In the course of this test, acoustic emission (AE) was used for real-time monitoring, and combined with micro visualization, the damage mechanism of composite multidirectional laminates was studied at multiple scales. Combining the AE detection results with micro visualization, it is found that the AE parameters and the damage to multidirectional laminates could realize a one-to-one correspondence. Through the study of the variation of the *RA* value, load, and strain energy release rate with the crack length, it is proved that the AE parameters can effectively characterize the initiation of delamination damage.

## 1. Introduction

Delamination damage is a key damage mode of composite laminate structures. Micro-splitting or macroscopic damage occurs in the damage process, which reduces the structural strength of laminates and finally leads to the complete failure of laminated structures. Type I interlaminar fracturing of composites is the most concerning problem for researchers because the initial energy release rate of delamination is lower than that of the shear mode. Some scholars have studied mode I interlaminar fracturing to determine the energy release rate of composites by producing a crack propagation effect [1,2]. Researchers have also considered the effects of environmental factors and defects in the manufacturing process on the strain energy release rate (*G*_I_) [3,4], as well as the effect of fiber laying direction on the delamination strain energy release rate [5,6,7]. The results show that the energy release rate of the adjacent interlaminar delamination of the laminate decreases with the increase in the interlaminar fiber angle [8]. Through the analysis of type I delamination of composite materials, it has been found that the type I delamination test depends on many factors, such as fiber direction, resin type, resin condition, temperature, and so on. Crack propagation is the final form of the failure mechanism in the interlaminar region in the process of delamination damage of laminates [9,10]. The failure degree of crack propagation in composite laminates is evaluated through direct observation or using load–displacement curves. Although the micro-damage behavior of the composite fiber matrix interface represents the main part of the delamination process, it is difficult to observe and study, since it involves meso and micro levels.

Acoustic emission (AE) technology has been widely used in the research on fracture mechanics and damage mechanics because of its advantages in non-destructive testing and the diagnosis of materials and structures [11,12,13]. A large number of studies [14,15] have shown that acoustic emission sources will be produced in the plastic deformation (PD) stage and the initiation stage of microcracks in the process of material damage, which is the reason why acoustic emission technology is widely used in material testing. Acoustic emission can detect the inherent crack defects in the material structure. When the stress and strain near the crack front change, the resulting elastic wave can be detected by the AET placed on the surface of the specimen. Therefore, how to use the acoustic emission method to quantitatively estimate the damage degree and inherent defects of material structure, and how to establish the relationship between acoustic emission signals and crack parameters, have always been research topics of great concern. Combining the strain energy release rate test using a double cantilever beam (DCB) with real-time acoustic emission monitoring can provide relevant information about the progressive damage evolution process of delamination of composite laminates. Most scholars use the conventional formula provided by the standard to estimate the strain energy release rate (*G*_Ic_), and only use acoustic emission as a tool to describe the delamination process of composite laminates rather than a research method to predict delamination damage of composite laminates [16,17,18]. Ndiaye et al. [19] classified AE signals with matching characteristics, established the relationship between the energy release rate and AE energy after the multivariate analysis of 49 parameters, and estimated *G*_Ic_ using AE information, but their method has some limitations in theory. M.G.R. Sause et al. [20] correlated the frequency of different acoustic emission signal types with the port microstructure of different composite samples, and they simulated and verified the acoustic emission signal of the typical failure mechanism of fiber-reinforced plastics. It is found that the damage characteristics extracted from the simulated signal are in good agreement with the test signal. The signal classification method is applicable to the failure analysis of carbon fiber and glass fiber under type I loading conditions. Most researchers use acoustic emission information to estimate some related material properties, such as ultimate strength, fracture toughness, residual strength after damage [21,22,23]. The regular classical method, based on empirical or semi-empirical formula, involves using mechanical parameters and acoustic emission information, respectively. How to use the acoustic emission signal as an independent parameter to predict or evaluate the properties of composites has been a very hot topic for researchers in recent years.

The damage model of composite laminates was discussed and studied in [24,25,26,27], but there is a lack of in-depth research on the dynamic damage evolution process and progressive failure mechanism of composite laminates with different interface fiber orientations. In this paper, type I interlaminar fracture toughness tests of carbon fiber laminates, with different interface fiber orientations, are carried out. The complementary nondestructive testing technology of acoustic emission (AE) and microscopic visualization is used to analyze the signal parameters and the damage mechanism of laminates. The results of acoustic emission and microscopic visualization are verified by the Rise Angle (*RA*) method. Based on the changes in material characteristics, the interlaminar failure behavior of laminates with different interface fiber orientations is discussed.

## 2. Materials and Methods

### 2.1. Material Preparation and DCB Test Procedure

In order to keep the bending stiffness of laminates within the range, the comprehensive effects of bending–torsion coupling, cantilever stiffness, and thermal residual stress were taken into account. A 24 layers laminate with stacking configuration of ${\left[{\left[\pm \nu /0_{5}\right]}_{AS}\right]}_{AS}$ with angles 0, 30, 40, 60 has been utilized [28,29]. The specific parameters of the manufacturing materials are listed in Table 1. According to the standard ASTM-D5528**(13)** [30], a 25 × 175 × 4.8 mm laminate sample was made, and a PTEE polytetrafluoroethylene film, with a thickness of 40 μm and a width of 20 mm, was inserted between the 12th layer and the 13th layer as a prefabricated crack. The laminate specimens, which were fabricated and met the strength requirements, were kept at a room temperature of 23 °C for more than 24 h in order to achieve the required performance index. After surface treatment, the surface of the laminate was cleaned with acetone solvent. Then the loading piano hinges, made according to standard ASTM D5528**(13)** [30], was glued to the upper and lower ends of the edge of the laminate sample with a mixture of polyamide and epoxy resin at 1:1, cured at room temperature for more than 8 h to meet the strength requirements under tensile load.

**Figure 1:**
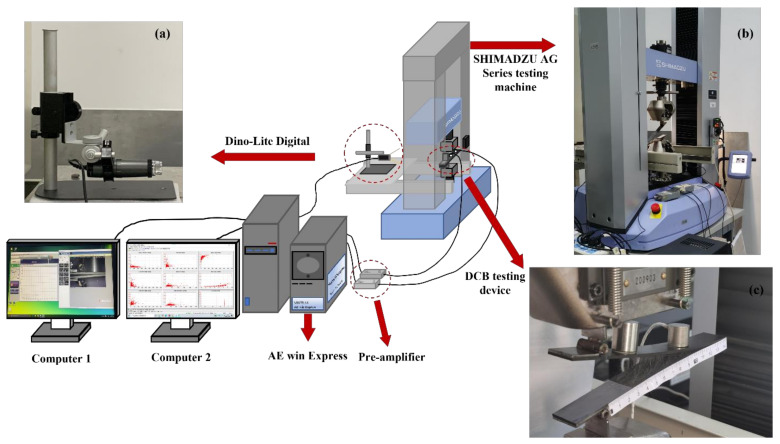
DCB testing device and AE data acquisition device (**a**) Dino-lite digital, (**b**) SHIMADZU AG Series universal electronic testing machine, (**c**) Laminate specimens and sensors.

According to the standard ASTM D5528**(13)** [30], the type I interlaminar fracture toughness test was carried out by using the SHIMADZU AG Series universal electronic testing machine (SHIMADZU Co., Ltd., Kyoto, Japan). The test sample was fixed to the electronic universal testing machine, with the loading piano hinges made according to the standard. As shown in Figure 1, the load was applied at a constant feed rate of 1 mm/min at a constant room temperature. The load–displacement, load–time, and stress–strain curves, fracture point stress, and other mechanical parameters of laminates were recorded by the Trape X data acquisition system. In the course of the experiment, a high optical zoom micro camera (Dino-lite digital) (AnMo Electronicswas Co., Ltd., 17F, No. 97, Sec. 4, ChongHsin Rd., Sanchong Dist., New Taipei City, 241 Taiwan) set up to focus on the edge of the laminate sample (as shown in Figure 1a), and the crack initiation and propagation were recorded.

### 2.2. AE Data Acquisition Device

Two acoustic emission sensors (α-series WSα SNAC88 and PACWD-286) (MISRAS Group, Inc., PAC, 195 Clarksville Rd, Princeton Jct, NJ 08550, USA) were installed on the upper surface of the laminate specimen, which were located at the edge of the prefabricated crack at 7 and 67 mm, respectively (as shown in Figure 1c), and the acoustic emission signals during the test were recorded in real time. The resonant frequency of the sensors was 125 kHz, and the optimum operating frequency ranges were 100–1000 kHz and 100–900 kHz, respectively. The threshold was 35 dB, and the gain was selected as the MISTRAS 2/4/6-AST preamplifier (MISRAS Group, Inc., PAC, 195 Clarksville Rd, Princeton Jct, NJ 08550, USA) of 40 dB. The sampling rate of AE data was 4 MSPS. According to ASTM E976-15 [31], we calibrated the sensor for the lead break test before each test. A special vacuum grease was used to cover the contact surface between the sensor and the laminate sample to achieve excellent acoustic coupling. The sensor was fixed with highly elastic glue in order to effectively avoid the influence of noise. Real-time AE data were collected by the MISTRAS AE win Express-16 channel system (MISRAS Group, Inc., PAC, 195 Clarksville Rd, Princeton Jct, NJ 08550, USA), as shown in Figure 1.

### 2.3. Strain Energy Release Rate

**Figure 2:**
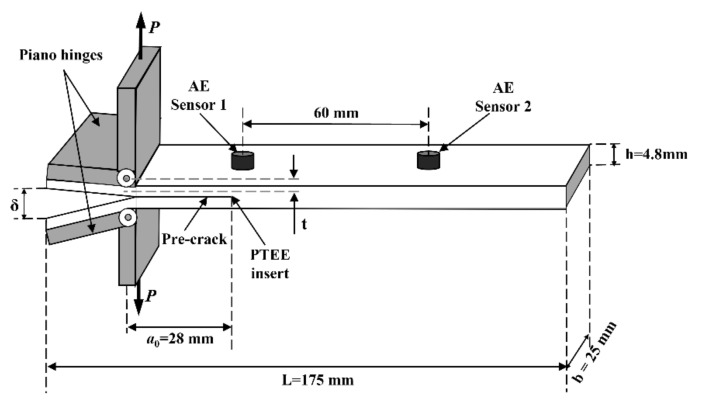
Geometry and nominal dimensions of specimen for mode-I DCB test.

For the delamination length of laminates with self-similar growth under constant displacement, the energy loss per unit sample width was the strain energy release rate [30]. The current standards do not provide any validity criteria for linear elastic fracture mechanic (LEFM) assumptions in multidirectional laminates. Therefore, the fracture resistance of multidirectional laminates is usually underestimated in the study of strain energy release rate [32]. According to ASTM D5528**(13)**, there are three different ways to calculate *G*_I_, which are modified beam theory (MBT), compliance calibration method (CC) and a modified compliance calibration method (MCC). In this work we have used the MBT method, because it has been shown to give the most conservative values of *G*_Ic_ in the literature [30]. The delamination toughness is obtained in terms of the critical strain energy release rate for beam-like specimens, employing data reduction methods, which are all based on the assumptions of linear elastic fracture mechanics (LEFM). After testing according to the standard, the beam theory expression for the strain energy release rate of a perfect build-in was used to calculate the *G*_I_ value, and the expression is as follows:

(1) $$G_{I}=\frac{3P\delta }{2ba}$$

where *P* is the applied load, *δ* indicates the corresponding displacement in the DCB tests, *b* is the width of the specimens, and *a* is the delamination length of the specimen, as shown in Figure 2.

**Figure 3:**
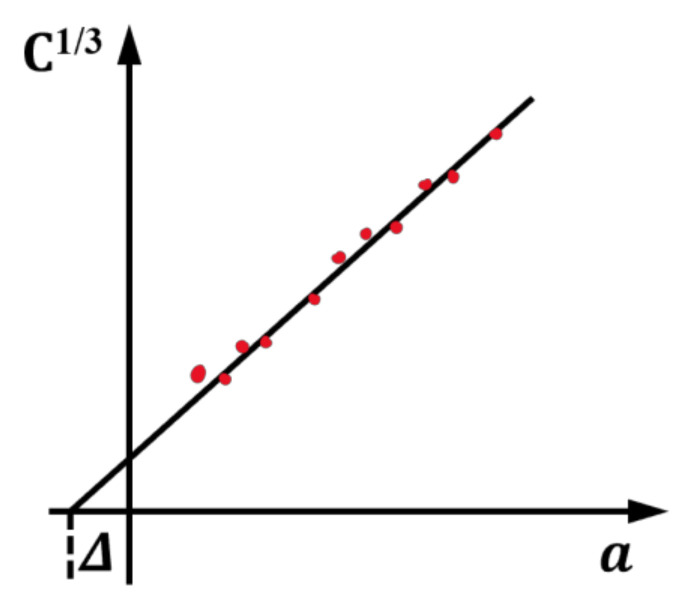
Modified beam theory.

In the actual DCB test, Formula (1) will overestimate *G*_Ic_ to some extent. According to the energy conservation law of dynamic crack propagation in fracture mechanics, the energy release rate *G*_I_ is greater than the crack resistance *G*_Ic_ [33,34,35,36,37]. In the process of crack propagation, the delamination front will rotate because the fiber is not completely built in. Therefore, Hashemi put forward the theory of equivalent crack length for the complex deformation of crack tip [38].

(2) $$a_{eff}=a+\left|\Delta \right|$$

where *a_eff_* is the equivalent crack length, and $\left|\Delta \right|$ is the correction value of crack length. The theory treats DCB as a slightly longer layer $a+\left|\Delta \right|$, where Δ can be determined by fitting the flexibility cube root *C*^1/3^ of the delamination length *a* during the test (Figure 3).

In this figure, *C* is the ratio of the load point displacement to the applied load, *δ*/*P*. When calculating *G*_Ic_ to account for the existence of the loading piano hinges, the standard ASTM D5528**(13)** [30] A1.1 modifies the large displacement effects shall be corrected by the inclusion of a parameter, *F*. The parameter *F* is introduced into the standard to explain the change in displacement effect with the increase in delamination length. This parameter, *F*, accounts for both the shortening of the moment arm as well as tilting of the end blocks.

(3) $$F=1-\frac{3}{10}{\left(\frac{\delta }{a}\right)}^{2}-\frac{3}{2}\left(\frac{\delta t}{a^{2}}\right)$$

where *t* is the distance from the loading piano hinge to the centerline of the front 12 layers of the laminate, shown in Figure 2:

(4) $$t=h/4+h_{p}$$

where *h* is the thickness of the laminate, and *h_p_* is the thickness of the loaded hinge. The equivalent crack length theoretical (Equation (2)) and the modified parameter *F* (Equation (3)) are substituted into Equation (1). According to Euler–Bernoulli beam theory, for a cantilever DCB specimen, the strain energy release rate is calculated as follows:

(5) $$G_{\mathrm{Ic}}=\frac{3P\delta }{2b(a+\left|\Delta \right|)}F$$

### 2.4. The Rise Angle Method

**Figure 4:**
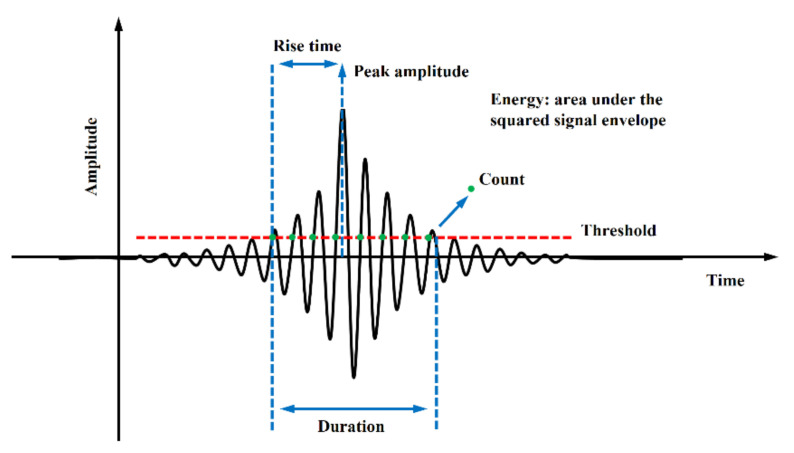
Common acoustic emission signal features (adapted from Ref. [41]).

Acoustic emission technology can be used to measure the stress waves caused by abrupt changes in some structures or materials, such as the initiation and propagation of microcracks [39]. There is a difference between AE signal and AE event. The release of each transient elastic wave will cause an AE event, which may be received by one or more AE sensors and form one or more hits. Therefore, an AE event corresponds to a rapid release of energy determined by one or more hits. The cumulative count of AE events reflects the total amount and frequency of AE events and can be used to evaluate the activity and location concentration of AE sources. Elastic waves, caused by changes in the internal sources of materials and structures propagate throughout the structure, and are received by sensors connected to the surface of the structure and converted into useful information in the AE system. The analysis of these acoustic emission events can provide a basis for the size, location, and cause of the damage type in the structure [40]. A typical AE signal and its main characteristic parameters are given in Figure 4. Among the AE parameters, the Maximum Amplitude and Duration of the signal are both a function of the fixed Threshold level. The area under the signal function that is linked with the Energy (AE Energy) dissipated in the damage process is also a significant parameter in the AE interpretation. The frequency component of the signal and its evolution, in the process of AE testing, provide a relevant basis for the analysis of the damage process. Therefore, by studying the evolution process of AE parameters and their combinations extracted from the signal, the information about the damage mechanism can be obtained.

The acoustic emission waveform has the characteristics of a certain fracture mode in the engineering field [42]. In the process of material damage, it can be measured that the tensile fracture velocity of the material is 60–70% of the acoustic emission longitudinal wave velocity, and the shear fracture velocity is 60–70% of the acoustic emission shear wave velocity. Based on this feature, the AE wave, which directly reflects the sound source-time function, can be established by corresponding to the crack type of the material. With the extension of delamination cracking of laminates, the rising amplitude of AE waveform gradually decreases, and the low-frequency components in the waveform will occupy the dominant position of frequency. Therefore, the Rise Angle (*RA*) method can be used to characterize the damage mechanism of crack propagation. The method is expressed as the ratio of the Rise Time (*RT*) to the Peak Amplitude (*A*) of the AE signal. The rise time of AE is the time that the signal crosses the threshold for the first time until the waveform reaches the maximum amplitude, and the peak amplitude is the maximum amplitude of the AE waveform [43]. The *RA* value expression is:

(6) $$RA=\frac{RT}{A}$$

where *RT* is the rise time of acoustic emission, which is expressed as μs, and *A* is the amplitude of the waveform, which is expressed as V.

*RA* values are usually used to classify failure mechanisms in concrete and other material structures, which can also be used to analyze the damage forms and failure characteristics of materials under loading. When the signal has a lower average frequency and a higher *RA* value, the signal is classified as a shearing mode. When the same signal has high average frequency and low *RA* value, it can also be classified as stretching mode [44]. The behavior was also noticed in the research carried out by Soulioti et al., so Soulioti et al. used *RA* value to study the signals in concrete materials containing different percentages of steel fiber and found that the *RA* value represents the tensile properties of the fracture specimen in the material [45]. In composite structures, the use of the *RA* value to analyze the signal is not directly related to the material failure mode. For example, the use of the *RA* value in composite laminates can identify the transition from matrix cracking to delamination and other damage mechanism characteristics [46,47,48]. De Sutter et al. also emphasized the importance of the *RA* value, especially in composites where the damage process is not directly related to the time distribution of acoustic emission signals [49].

## 3. Results and Discussion

### 3.1. Energy Release Rate of Laminates with Different Interface Fiber Orientation

**Figure 5:**
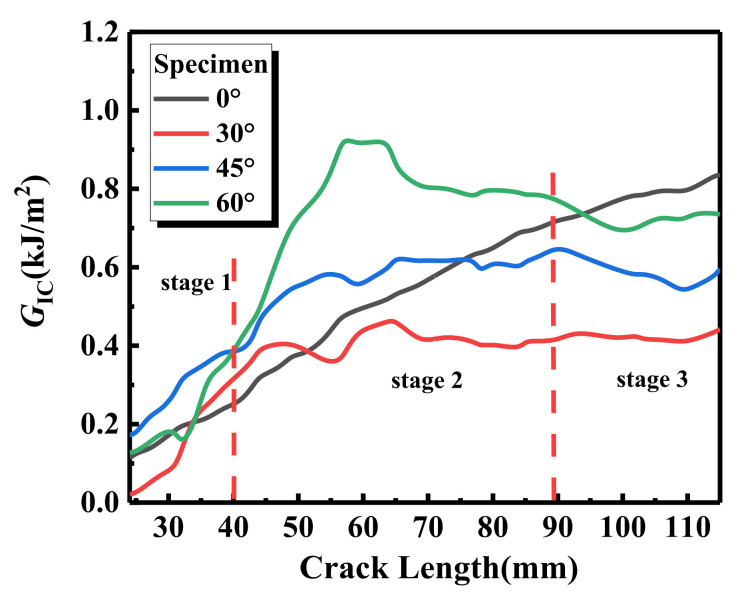
Energy release rate for different interface fiber orientations.

According to Equation (5), the strain energy release rates of laminates with different interface fiber orientations, in the process of crack initiation and propagation, are obtained, as shown in Table 2 and Table 3. The energy release rate curve can be divided into three stages, as shown in Figure 5. With the crack initiation and propagation of laminates, the strain energy release rates of laminates with different interface fiber orientations show an upward trend and resistance fracture behavior. In the first stage, the slope of the overall ascending trend is larger, and the load of the sample with unidirectional (UD = 0°) laminate is the largest, while the energy release rate is the lowest. When the crack propagates to the second stage, except for the UD laminate, the energy release rate of the other three paved specimens decreases slowly when the crack length reaches 50~60 mm. The energy release rates of the four interface fiber orientations are 0.4767, 0.3758, 0.5764, and 0.9308 kJ/m^2^, respectively, as shown in Table 3. In the third stage, except for the UD laminate, the energy release rates of the other three laying angle specimens showed a downward trend, and increased slowly after the crack extended to 110 mm. At this time, the energy release rates of the four interface fiber orientation specimens were 0.7852, 0.4276, 0.5795, and 0.6984 kJ/m^2^, respectively. The effective delamination extension to correct for the rotation of DCB arms at the delamination front caused by crack propagation was 0.021095, 0.0041697, 0.007669, and 0.00461664 mm, respectively. When the samples with four interface fiber orientation reached the maximum load (111.34 ± 0.14, 66.28 ± 0.12, 80.87 ± 0.32, 112.21 ± 0.07 N), the energy release rate fluctuated to different degrees, the load curve showed a downward trend, and the highest displacement was 6.29 ± 0.13, 3.78 ± 0.01, 3.72 ± 0.29, and 0.98 ± 0.15 mm, respectively. MBT method considers a linear elastic behavior and assumes that the nonlinear effects are confined to a small-scale fracture process zone (FPZ) ahead of the crack tip. When extensive nonlinearity occurs, the small scale FPZ assumption breaks down, and the LEFM based results can be biased [32].

**Figure 6:**
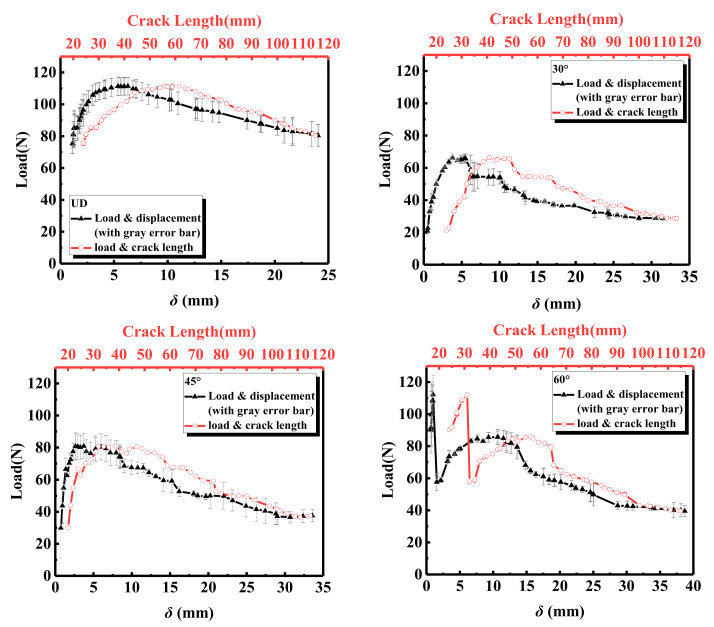
Variation of load for different interface fiber orientations with displacement and crack length.

Figure 6 shows the load as a function of displacement and delamination length for laminates with different interface fiber orientations. The standard error of triplicate experiments is also indicated by the gray error bar in the figure. It can be seen that, at similar displacements, the unidirectional laminate has the highest load, which means that the crack in the unidirectional (UD = 0°) specimen requires a larger load to propagate. Due to the existence of prefabricated cracks, the tensile load-opening displacement of the laminates, with different interface fiber orientations, showed a linear relationship in the load rising stage. The load of the 60° interface fiber orientation showed a rapid increase, and then a sudden decrease, in the opening displacement and the initial stage of cracking, which was mainly caused by the bonding between the loading piano hinge and the laminate. When the crack length reaches the delamination front of the prefabricated crack, the load and opening displacement of the laminates with different interface fiber orientations show nonlinear characteristics. When the maximum load is reached, the destabilization expansion of delamination damage causes the load of the fiber-oriented laminates at different interfaces to show a downward trend. Except for the UD specimen, the other three interface fiber-oriented laminates show anti-fragmentation bending effects. Due to the uneven layered growth of cracks along the specimen, the load curve exhibits multiple fluctuations of rising and falling.

### 3.2. Analysis of Acoustic Emission and Damage Mechanism of Laminated Plates with Different Interface Fiber Orientations

**Figure 7:**
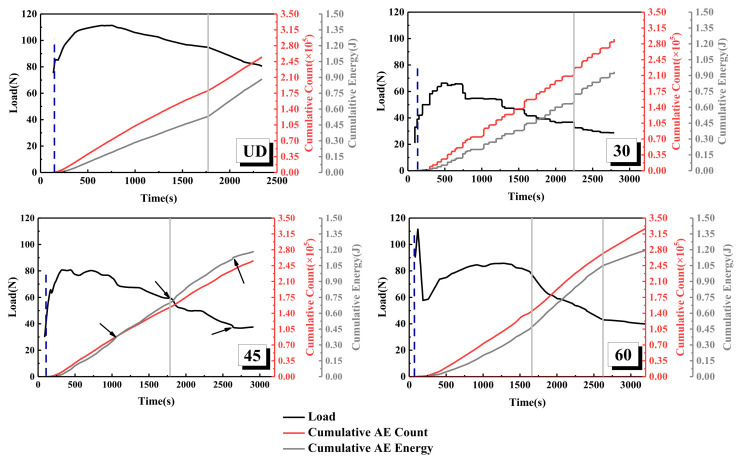
Load, cumulative AE energy, and count versus time for different interface fiber orientations.

The variation curves of the load, cumulative acoustic emission energy, and cumulative count of laminates, with four different interface fiber orientations, are shown in Figure 7. Among them, the load of the UD laminate is the highest, and the crack initiation needs a higher load. Compared with the UD laminate, the cracking loads of the 30° laminate and the 45° laminate are relatively lower, mainly because the internal fiber exists at an angle, which leads to the decrease in bearing capacity and uneven force in the laminate. The loading curve of the 60° laminate experiences a sudden increase and decrease during the period of 0~500 s, which is mainly due to the high bonding quality between prefabricated cracked PTEE film and laminate, which leads to high strength at the initial cracking stage. As a result, the test duration of the 60° laminate is the longest. The cumulative AE energy and cumulative counting curves of the laminates, with four different interface fiber orientations, have the same trend. With the decrease in load, the rising slopes of the cumulative AE energy curve and cumulative counting curve become larger. The position marked by the gray line in Figure 7 can clearly show the steepness of the curve. The dependence of the cumulative AE energy on the tensile stress is mainly determined by the load and the relevant energy released by the AE event. Therefore, the sudden change in the slope of the cumulative AE energy curve indicates that another damage mechanism has occurred at this stage [50,51,52]. The cumulative AE energy of the UD laminate mainly comes from matrix cracking, and the crack propagation always propagates horizontally along the center line of the pre-crack at an almost constant rate, and the cumulative AE energy and cumulative counting curves are relatively smooth. The cumulative AE energy and cumulative count of the 30° laminate show a multi-step phenomenon, in which the instantaneous energy release rate is accompanied by the generation of higher amplitude AE signals, and the cumulative energy and time curve of acoustic emission has a multi-stage ladder consistent with [53]. This phenomenon of multi-step ladders reflects the essence of the sudden increase in the energy release rate caused by fiber fracture and the unstable propagation of matrix cracks.

It can be seen from Figure 7 that the strain energy release rate of the 30° laminate is the lowest, and the highest point can bear the load of 66.28 ± 0.12 N. The fiber of the 30° laminate is angled, so the stress of crack propagation fiber is uneven under tension. The decrease in interlaminar shear strength leads to the fracture of a large number of interlaminar fibers after the bridging phenomenon, and the crack propagates suddenly. The cumulative AE energy and cumulative counting curves of the 45° laminate have three inflection points in Figure 7—with the sudden drop in load at about 1000 s, the cumulative AE energy suddenly increases, and the load decreases from 79 to 65 N; then, at the second inflection point of 1700 s, the load decreases from 60 to about 50 N, and the cumulative AE energy increases again; until about 2700 s, when the load has an upward trend again, and the cumulative AE energy has a slow downward trend. This also shows that the tensile stress of the 45° laminate reaches the highest point at the third inflection point, which is the early stage of instability. The cumulative AE energy and cumulative counting curves of the 60° laminate also have two inflection points at about 1600 and 2600 s. Combined with the changing trend of the energy release rate curve of Figure 5, the energy release rate of the 60° laminate at the two inflection points has a downward trend, and the load also shows a downward trend; on the contrary, the cumulative AE energy has a sudden upward trend.

**Figure 8:**
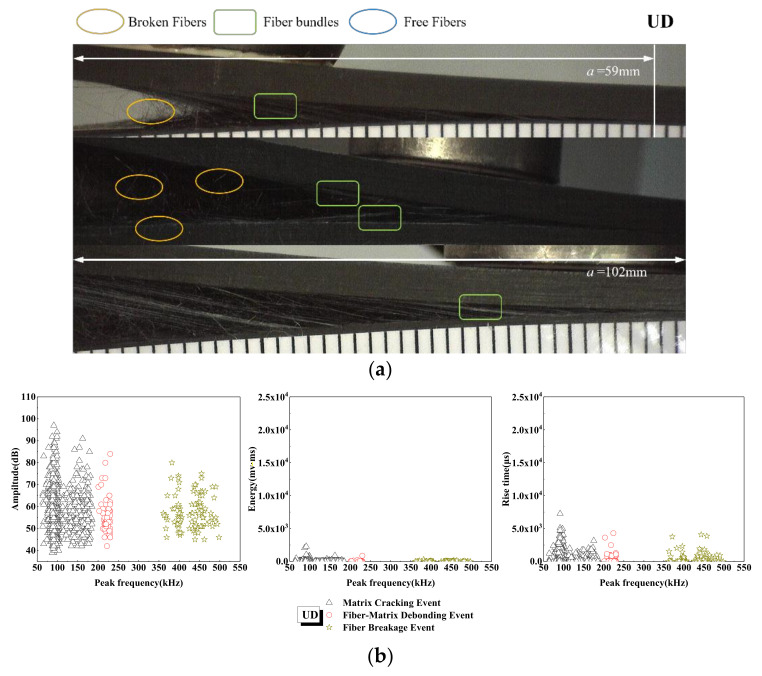
Damage mechanism for the UD interface fiber orientation and evolution analysis of AE characteristics (**a**) Microscopic interface of UD laminate, (**b**) Evolution analysis of AE characteristics of UD laminate.

Different frequency ranges of acoustic emission signals are related to different failure modes of composites [54,55,56]. It can be seen from Figure 8 that the damage mechanism of the UD laminate, based on frequency distribution, is mostly concentrated in the matrix cracking event, and the amplitude is in the AE signal of 35~100 dB. With the increase in tensile load, the debonding signal of the fiber matrix is higher than that of cracking, and then a large number of signals appear in the stage of fiber fracturing. In addition, the occurrence time of interfacial debonding is later than that of cracking, mainly because the damage evolution processes of the two failure mechanisms are different. As the support, the matrix first contacts and bears the tensile load, so the crack at the interface between the resin and the fiber bundle propagates under the action of stress concentration, so the fiber bundle gradually bears the main load with the increase in sample load and tensile deformation. In the process of delamination instability propagation, the crack propagation leads to the interface debonding between the fiber in the crack initiation zone and the matrix. It can be seen from the microscopic interface of Figure 8 that the crack in the UD laminate always propagates slowly along the pre-crack layer, and the damage mechanism is mainly concentrated in the stage of matrix cracking and fiber fracture. In addition, through the variation of AE energy and rise time with peak frequency in Figure 8, it can be seen that in the three damage events, the amplitude and rise time of debonding and fiber fracture are lower than that of matrix cracking. The overall damage of UD laminate is small, and the energy is low, which further shows that the signal generation phenomenon is consistent with the cumulative AE energy and the cumulative counting damage evolution, and the crack propagation is uniform and slow, and the damage accumulation is mainly concentrated in the delamination tip area.

**Figure 9:**
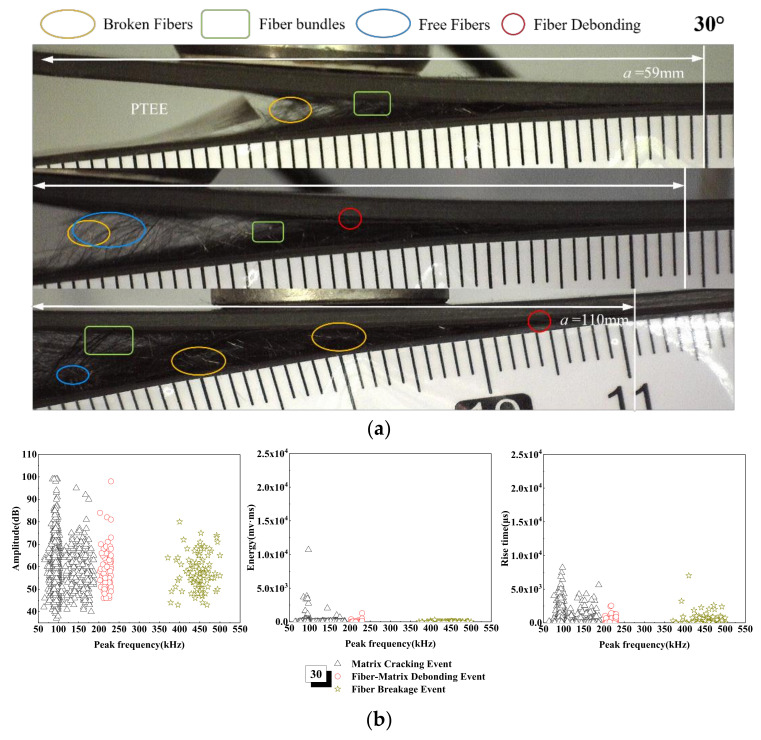
Damage mechanism for the 30° interface fiber orientation and evolution analysis of AE characteristics (**a**) Microscopic interface of 30° laminate, (**b**) Evolution analysis of AE characteristics of 30° laminate.

The damage mechanism of the 30° laminate is concentrated in matrix cracking and fiber fracture events, and the acoustic emission signals with amplitudes in the range of 35~100 dB, is increased obviously, as shown in Figure 9. The variation of the AE amplitude, energy and rise time with peak frequency are not different from that of the UD laminate, but with the increase in tensile load, the peak frequency is approximately between 50 and 200 kHz, which means that the energy of 10,000 mv·ms appears in the cracking stage of the matrix. The peak frequency is within the range of 200~250 kHz—that is, a signal as high as 100 dB appears in the stage of fiber debonding. This is due to the aggravation of matrix damage. Because of the angle of the fiber, the fiber fracture is bundled rather than filamentous, and the sudden rupture of the fiber bundle will lead to the increase in signal energy. From the microscopic interface of Figure 9, it can be seen that, when the crack extends to 59 mm, there are debonding fiber bundles and a large number of broken fibers come out, and the interfacial debonding occurs after cracking. Additionally, due to the existence of the fiber laying angle, a large number of fibers break in the shape of bundles in the cracking process, the cracks are uneven, and the laid fibers are debonded from other layers in the process of the crack extending.

**Figure 10:**
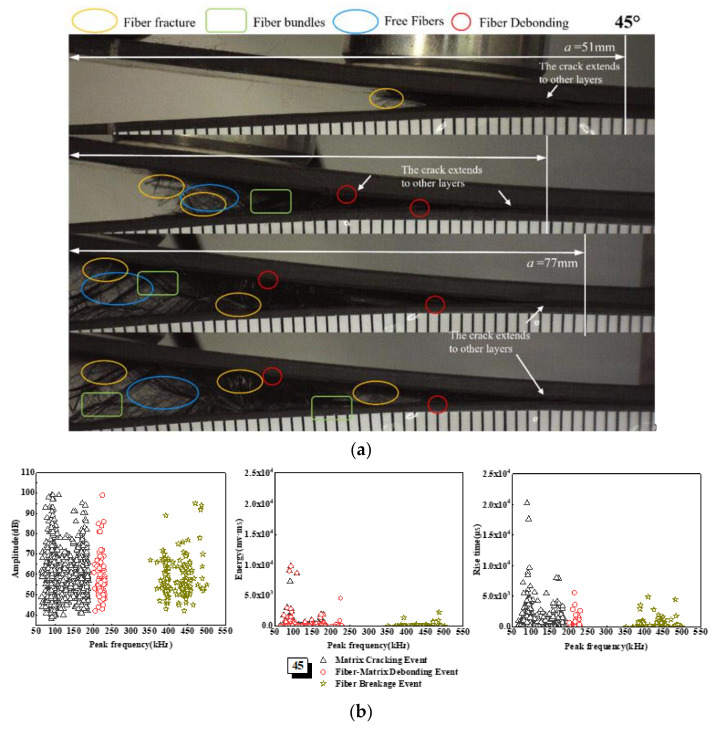
Damage mechanism for the 45° interface fiber orientation and evolution analysis of AE characteristics (**a**) Microscopic interface of 45° laminate, (**b**) Evolution analysis of AE characteristics of 45° laminate.

The amplitude, energy, and rise time of AE of the 45° laminate change with the peak frequency—that is, the three damage mechanisms are significantly higher than those of the UD and 30° laminates, as shown in Figure 10. From the microscopic interface, it can be seen that there are many damage mechanisms in the failure forms of the 45° laminate, such as matrix cracking, bundle cracking, interfacial debonding, interfacial slip, fiber fracture, fiber bundle fracture and fiber pull-out. Therefore, in the form of instability and failure, acoustic emission produces a large number of signals with a wide range of distribution. From the variation diagram of energy with peak frequency, it can be seen that there is a phenomenon of signal superposition in the range of 50~250 kHz, and there is a partial overlap between matrix cracking and fiber matrix debonding signals. This is mainly due to the superposition of damage modes when the specimen fails; the complex AE signal superposition phenomenon will focus on instability failure and multiple damage mechanisms [57]. The transverse crack propagation generally occurs relatively straight along the position of the pre-crack under high tensile stress. As can be seen from the microscopic interface in Figure 10, with the delamination crack of the specimen, the crack in the bundle crack area will be closer. Additionally, with the gradual increase in the load, the angular fiber leads to the slip of the crack tip, resulting in serious interfacial debonding of the fiber matrix (such as *a* = 77 mm in Figure 10). As a result of interface slip and fiber bundle fracture, the fibers are easily pulled out, and a large number of fibers are pulled out at the edge of the sample.

**Figure 11:**
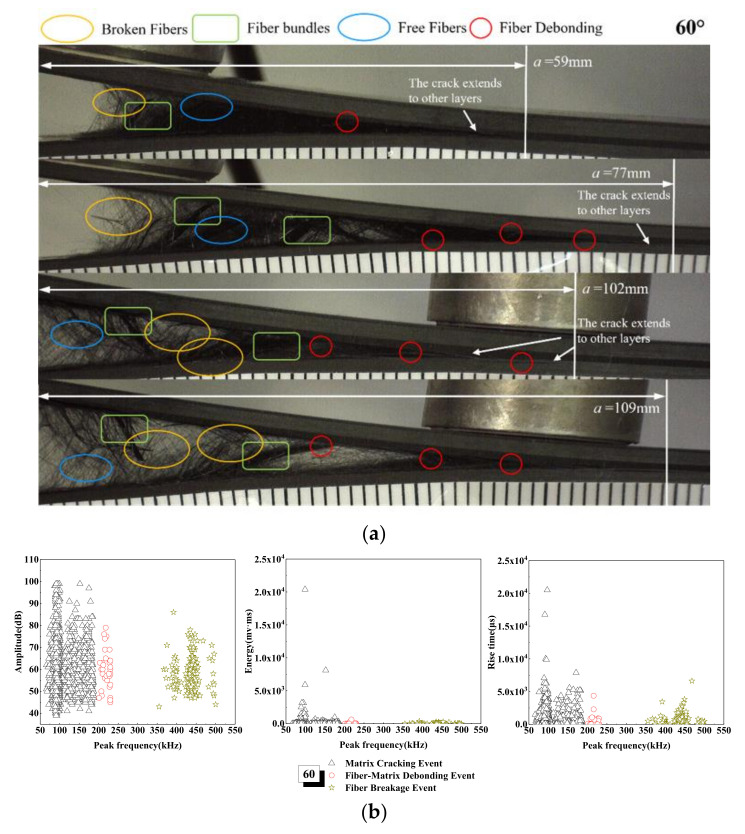
Damage mechanism for the 60° interface fiber orientation and evolution analysis of AE characteristics (**a**) Microscopic interface of 60° laminate, (**b**) Evolution analysis of AE characteristics of 60° laminate.

The failure morphology of the 60° laminate is similar to that of the 45° laminate, but the former slip is more serious. The variations of the AE amplitude, energy, and rise time with peak frequency of the 60° laminate are shown in Figure 11. In the matrix cracking stage—that is, in the range of peak frequency of 50~200 k Hz—the amplitude range of damage does not change, but the number of signals increases, and the AE energy is as high as 20,000 mv·ms. This is mainly due to the fact that, under the action of progressive tensile load, the fiber bundle leads to interface slip at an angle, resulting in sudden failure, as can be seen from the strain energy release rate curve in Figure 5. From the microscopic interface in Figure 11, we can see that there are more fiber pull-out damage modes in the 60° laminate, and when delamination length is 59 mm, fiber bundle fracture and interface slip occur. When delamination length reaches 77 and 102 mm, a large number of fibers are pulled out from the matrix in bundles and broken, and the phenomenon of fiber bridging is serious. Due to the nature of fiber bridging, when there is a crack between layers, the fiber at the laying angle will be bridged on the two surfaces of the interlayer crack, and when the crack extends and expands, gradually, the fiber will gradually bend and experience tensile deformation. The interaction between the fiber and the matrix will cause fiber/matrix debonding and part of the matrix to fall off, which will delay the delamination growth, as shown in Figure 6. Therefore, the delamination failure time of the 60° laminate is the longest under load [58].

**Figure 12:**
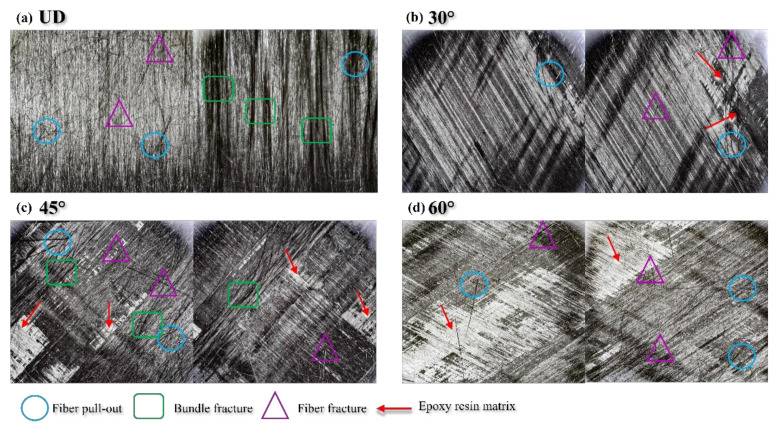
Interface after delamination failure of specimens with different fiber orientations (**a**) UD, (**b**) 30°, (**c**) 45°, (**d**) 60°.

In addition, through the delamination failure interface of four interface fiber orientations (Figure 12), it can be found that the damage mechanism of the UD laminate is concentrated in fiber pulling out, fiber fracture, fiber bundle fracture, and so on. As for the 30° laminate, due to the laying angle, as can be seen in Figure 12b, after the fiber is pulled out and broken, part of the epoxy resin matrix leaks out. The transverse interface damage of the 45° laminate is more serious and complex, the fiber is pulled out and broken in the shape of the wire bundle, the bulk resin matrix is exposed, there are holes in the pull-out place of the fiber, and a large number of broken fibers appear in the left and right edges and weak areas in the middle of the specimen. The fiber bridging phenomenon of 60° laminate is serious, so a large area of epoxy resin matrix can be seen on the damaged cross section; the laid fiber is pulled out and broken in the shape of wire bundle, and there are a large number of broken fibers left on the failure interface of the specimen.

Through the observation and analysis of acoustic emission parameters, energy release rate, and microscopic visualization, it can be found that the various damage modes observed from microscopic visualization are consistent with the results analyzed from the point of view of acoustic emission signals. The results show that the cross-verification of acoustic emission and microscopic visualization can effectively explain the progressive evolution behavior of laminates under delamination damage, and acoustic emission can characterize the evolution law of the delamination damage of carbon fiber laminates to a certain extent.

### 3.3. The RA Values of Laminates with Different Interface Fiber Orientation

**Figure 13:**
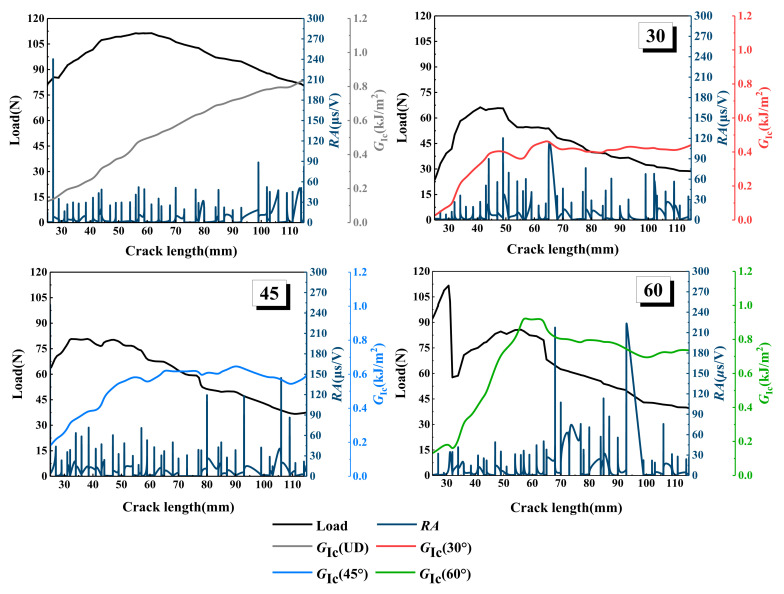
Load, energy release rate, and RA value versus the delamination length for different interface fiber orientations.

The comparative analysis of the above acoustic emission analysis results and energy release rate shows that the most directional trend is that the *RA* value will increase with the increase in load. The variation curves of *RA* value, energy release rate, and load, with crack length of the different interface fiber orientations, are shown in Figure 13. It can be seen from the figure that the strain energy release rate of the UD laminate is low at the initial stage of loading, and the *RA* value is as high as 240 μs/V at 79 N. Then, the *RA* value is always lower than 60 μs/V until the crack extends to 100 mm, when the *RA* value suddenly increases to 90 μs/V. According to the definition of *RA* value, a higher *RA* value indicates that the laminate has transverse cracking during delamination failure, which confirms the damage mechanism of the UD laminate in Figure 7. The crack initiation of type I damage is initially caused by triggering delamination, and then, the crack propagates slowly; the damage is mainly concentrated in the crack tip area, and the transverse cracking gradually reaches saturation until the specimen fails. The fluctuation of the *RA* value of the 30° laminate is consistent with the increase in energy release rate and load fluctuation; when the cracking length is 40~50 mm, the *RA* value increases to 90 and 120 μs/V, and the energy release rate increases to 0.4 kJ/m^2^. When the delamination crack increases to 50 mm, the load on the specimen is 66.28 ± 0.12 N, which is the maximum load of 30° laminate, and the maximum *RA* value is produced in the loading step, which indicates that the transverse crack is close to or reaches saturation. Fiber debonding occurs here, which verifies the results of microscopic visualization and AE signal analysis in Figure 9. The *RA* value step of the 45° laminate is different from that of the UD and 30° laminates, which mainly occurs during the delamination length from approximately 80 to 110 mm. The energy release rate of the 45° laminate increases gently, and the *RA* value increases obviously at the position of the energy release rate fluctuation and loading step. With the decrease in the load, the *RA* value increases significantly, which is mainly due to the fiber bridging phenomenon of the 45° laminate with the crack, which leads to the interlaminar damage being concentrated in the pulling out and debonding of the fiber bundles in the later stage of loading, leading to serious shear cracking, so a higher *RA* value occurs during failure, which is consistent with the results found in Figure 10. The *RA* value of the 60° laminate is the highest among the four kinds of laminates with different interface fiber orientations; it can be seen from Figure 13 that, when the delamination length is 70 mm, the strain energy release rate decreases steeply, the load decreases from 80 to 65 N, and the *RA* value increases to 210 μs/V. Consistent with the conclusion in Figure 11, the interface slip occurs at the crack tip because there are a large number of fibers to be pulled out in bundles, and sudden debonding after delamination propagation is delayed, resulting in a large amount of AE energy and a large *RA* value.

The above parameter analysis and microscopic visualization multi-scale comparative analysis show that certain acoustic emission parameters can be used to characterize the damage mechanism and damage initiation of multidirectional laminated composites.

## 4. Conclusions

In this paper, according to standard ASTM D5528**(13)**, DCB tests were carried out on CFPR composite laminates with four different interface fiber orientations. Through the analysis of the accuracy of the prediction of delamination damage initiation, according to the energy release rate in the process of delamination expansion of multidirectional laminates, the DCB test results were analyzed by using real-time observed AE event parameters and microscopic visualization. Combined with the verification of the analysis results by the *RA* value, the following conclusions can be drawn:

With the initiation and propagation of cracks, the energy release rates of CFPR laminates with four interface fiber orientations are different. The energy release rate of the UD laminate increases with the increase in crack length. With the increase in crack length, the energy release rate of the 30°, 45°, and 60° laminates increased at first and then decreased slowly. When the samples with different interface fiber orientations reach the maximum load, the strain energy release rate will fluctuate in different degrees, and the load curve shows a downward trend.

The changes in the cumulative AE energy and cumulative count of laminates with four interface fiber orientations with time are analyzed. It is found that the UD laminate has the strongest bearing capacity, while the 30°, 45°, and 60° laminates have a lower load bearing capacity and uneven stress due to the angle of internal fibers in laminates. The cumulative AE energy and cumulative counting curve of laminated plates with four interface fiber orientations have the same trend in general, and the rising slopes of the cumulative AE energy curve and the cumulative counting curve increase with the decrease in load. Among them, the cumulative AE energy and cumulative count of the 30° laminate show a multi-step phenomenon.

According to the multi-scale analysis of the damage mechanism of multidirectional laminates based on acoustic emission signals. It is found that the damage mechanism of the UD laminate is mostly concentrated in the matrix cracking event, the overall damage amount is small, the energy amplitude is low, and the crack propagation is uniform and slow. The damage mechanism of the 30° laminate is mainly focused on matrix cracking and fiber fracture events, while the changes in acoustic emission amplitude, energy, and rise time with peak frequency are not different from those of UD laminate. There are many kinds of damage mechanisms in the failure forms of the 45° laminate. In the form of instability failure, acoustic emission produces a large number of signals with a wide range of distribution, and the damage superposition mode appears when the energy in the 50~250 kHz interval changes with the peak frequency. The failure morphology of the 60° laminate is similar to that of the 45° laminate, but the interface slip is more serious. Because of the angle of fiber bundle, the damage amplitude range remains unchanged, the number of signals increases, and the acoustic emission energy is as high as 20,000 mv·ms.

Based on the comparative analysis of the *RA* value, energy release rate, and load of multidirectional laminates, it is found that each increase in the *RA* value is closely related to the damage mechanism of laminates, and this is verified with the energy release rate curve; it is thus confirmed that the AE parameter is feasible for assessing the damage mechanism and initiation of laminated composites.

## Figures and Tables

**Table 1:** Material parameters of carbon fiber laminate (Unidirectional plate).

Material Parameters	T700 Carbon Fiber	Carbon Fiber Bundle Yarn	Epoxy Resin Matrix TDE-85#
Tensile strength of bundle (MPa)		4035	
Tensile modulus of bundle yarn (GPa)		210.64	
0° tensile strength (MPa)		2178	
0° tensile modulus (GPa)	132		
0° compressive strength (MPa)	1039		
0° compression modulus (GPa)	131		
Poisson’s ratio (%)	0.29		
90° tensile strength (MPa)	24		
90° tensile modulus (GPa)	11		
90° compressive strength (MPa)	168		
90° compression modulus (GPa)	13		
Bending strength (MPa)	1554		
Flexural modulus (GPa)	110		
Shear strength between layers (MPa)	81		
45° shear strength (MPa)	19		
45° shear modulus (GPa)	5.2		
Tensile strength (MPa)			60
Tensile modulus (GPa)			3.7

**Table 2:** The parameter of different interface fiber orientations (*P*_max_).

Simple Code	*P*_max_/N	*δ*/mm	Δ/mm	*G*_Ic_/kJ/m^2^	*h*/mm
UD = 0°	111.34 ± 0.14	6.29 ± 0.13	0.021095	0.46 ± 0.015	4.7 ± 0.15
30°	66.28 ± 0.12	3.78 ± 0.01	0.0041697	0.32 ± 0.019	4.7 ± 0.05
45°	80.87 ± 0.32	3.72 ± 0.29	0.007669	0.38 ± 0.037	4.8 ± 0.02
60°	112.21 ± 0.07	0.98 ± 0.15	0.00461664	0.18 ± 0.05	4.8 ± 0.15

In the table, *P*_max_ indicates the maximum applied load during the DCB test, and *δ* indicates the corresponding displacement in DCB tests at *P*_max_. *h* is the thickness of the specimens, while Δ is the effective delamination extension to correct for rotation of DCB arms at delamination front, and *G*_Ic_ is the Mode I strain energy release rate at *P*_max_.

**Table 3:** The parameter of different interface fiber orientations at different stages.

Stage 1	Simple Code	*P*/N	*δ*/mm	*G*_Ic_/kJ/m^2^
	UD = 0°	99.8004 ± 0.008	2.4243 ± 0.0018	0.2416
	30°	63.7001 ± 0.014	3.3145 ± 0.002	0.2947
	45°	80.8795 ± 0.001	3.7247 ± 0.001	0.3872
	60°	73.6131 ± 0.0397	3.4008 ± 0.0021	0.3454
Stage 2	Simple code	*P*/N	*δ*/mm	*G*_Ic_/kJ/m^2^
	UD = 0°	111.111 ± 0.0159	5.6338 ± 0.0023	0.4767
	30°	54.7648 ± 0.0001	7.0838 ± 0.0023	0.3758
	45°	74.0131 ± 0.0001	8.5247 ± 0.0001	0.5764
	60°	84.9009 ± 0.0053	11.5402 ± 0.002	0.9308
Stage 3	Simple code	*P*/N	*δ*/mm	*G*_Ic_/kJ/m^2^
	UD = 0°	87.7734 ± 0.028	18.6511 ± 0.0042	0.7852
	30°	32.0064 ± 0.0088	24.2292 ± 0.0041	0.4276
	45°	41.50073 ± 0.0001	26.2656 ± 0.0001	0.5795
	60°	42.7421 ± 0.0114	30.1013 ± 0.0045	0.6984

## Data Availability

Data can be provided upon request from the correspondent author.
